# Various types of autoimmunological diseases of thyroid gland

**DOI:** 10.1186/1756-6614-6-S2-A55

**Published:** 2013-04-05

**Authors:** Stanisław Sporny

**Affiliations:** 1 Department of Dental Pathology, Medical University of Lodz, Poland

## 

Thyroid gland disease of autoimmune origin presents wide spectrum of pathologies, Graves’ disease from one side and chronic thyroiditis with fibrosis and atrophy of gland parenchyma from the other one. Among them the highest number of misunderstandings is observed in pathological reports of chronic thyroiditis in pre-operative cytological as well as in post operative histological examinations. There are numerous classifications of chronic thyroiditis, however they are not widely accepted. Histoclinical classification which correlates elements of microscopic examination with clinical course of the disease seems to be ideal but still does not exist. FNAB of thyroid gland is a method of the greatest importance in the diagnosis of chronic thyroiditis however that fact is not widely understood in every day routine of clinicians and pathologists.

